# Kallistatin prevents ovarian hyperstimulation syndrome by regulating vascular leakage

**DOI:** 10.1111/jcmm.17491

**Published:** 2022-07-21

**Authors:** Jianfang Huang, Yuling Mao, Quanxin Li, Honghai Hong, Ni Tang, Xiangjin Kang, Yuling Huang, Jianqiao Liu, Qing Gong, Yachao Yao, Lei Li

**Affiliations:** ^1^ Key Laboratory for Major Obstetric Diseases of Guangdong Province Department of Obstetrics and Gynecology, Center for Reproductive Medicine The Third Affiliated Hospital of Guangzhou Medical University Guangzhou China; ^2^ Department of Reproductive Medicine Ganzhou People's Hospital Ganzhou Jiangxi China; ^3^ Department of Biochemistry and Molecular Biology, GMU‐GIBH Joint School of Life Sciences Guangzhou Medical University Guangzhou China; ^4^ Department of Clinical Laboratory The Third Affiliated Hospital of Guangzhou Medical University Guangzhou China; ^5^ Department of Laboratory Medicine Guangdong Second Provincial General Hospital Guangzhou China; ^6^ Key Laboratory for Reproductive Medicine of Guangdong Province The Third Affiliated Hospital of Guangzhou Medical University Guangzhou China

**Keywords:** hCG, Kallistatin, OHSS, vascular leakage, VEGF

## Abstract

Angiogenesis and increased permeability are essential pathological basis for the development of ovarian hyperstimulation syndrome (OHSS). Kallistatin (KS) is an endogenous anti‐inflammatory and anti‐angiogenic factor that participates in a variety of diseases, but its role in OHSS remains unknown. In this study, treating a human ovarian granulosa‐like tumour cell line KGN and human primary granulosa cells (PGCs) with human chorionic gonadotropin (hCG) reduced the expression of KS, but increased the expression of VEGF. Furthermore, we found that KS could attenuate the protein level of VEGF in both KGN cells and human PGCs. More interestingly, we observed that exogenous supplementation of KS significantly inhibited a series of signs of OHSS in mice, including weight gain, ovarian enlargement, increased vascular permeability and up‐regulation of VEGF expression. In addition, KS was proved to be safe on mice ovulation, progression of normal pregnancy and fetus development. Collectively, these findings demonstrated that KS treatment prevented OHSS, at least partially, through down‐regulating VEGF expression. For the first time, these results highlight the potential preventive value of KS in OHSS.

## 1 INTRODUCTION

Ovarian hyperstimulation syndrome (OHSS) is one of the main complications of assisted reproductive technology (ART) and is commonly induced by aggressive controlled ovarian hyperstimulation (COH) to achieve mature oocytes in women with infertility issues. OHSS is characterized by enlargement of ovaries and extravasation of intravascular fluids to the third space, thus leading to ascites and oedema. In severe cases, it may even be life‐threatening.^1^ Therefore, prevention of OHSS is very important.

Angiogenesis and vascular hyperpermeability have been reported to be the pathological basis of OHSS, which are induced by the release of vasoactive and angiogenic substances from the ovary, including vascular endothelial growth factor (VEGF).^2^ Previous studies have confirmed that OHSS was associated with a higher amount of serum and follicular fluid VEGF. Over‐secretion of ovarian VEGF is usually triggered by human chorionic gonadotropin (hCG) and binding to VEGF receptor 2 (VEGFR2) on endothelial cells, which leads to clinical manifestations of OHSS.^3^, ^4^, ^5^, ^6^ Thus, VEGF appears to be the key element in the development of OHSS. Targeting VEGF may be an effective strategy to both prevent and treat OHSS.

Kallistatin (KS), also known as kallikrein‐binding protein (KBP), belongs to the serine proteinase inhibitor (serpin) superfamily. KS participates in a series of pathophysiological processes with such as hypertension, inflammation, diabetes, optic nerve injury and angiogenesis.^7^ KS is reported to display both anti‐angiogenic and pro‐angiogenic effects via its heparin‐binding site and active site, respectively. Those two totally inverse effects are embodied in inhibiting angiogenesis by acting on endothelial cells, while promoting neovascularization by acting on endothelial progenitor cells.^8^ An increasing number of studies have shown that KS delayed tumour‐associated angiogenesis by decreasing VEGF expression.^9^, ^10^, ^11^ Moreover, KS can down‐regulate VEGF expression to improve vascular leakage and alleviate manifestations in diabetic retinopathy (DR) rat model.^12^, ^13^ However, its effect on OHSS has not yet been studied. We hypothesized that KS may have a preventive effect on OHSS via modulating VEGF expression.

## 2 METHODS AND MATERIALS

### 2.1 Reagents

The following reagents were used: Dulbecco's modified Eagle's medium/Ham F12 1:1 (DMEM‐F12) (Corning, NY, USA); penicillin and streptomycin (Thermo Fisher Scientific, MA, USA); foetal bovine serum ([FBS] Thermo Fisher Scientific); anti‐Kallistatin (ab187656; Abcam, Cambridge, UK); anti‐VEGF (sc‐53462; Santa Cruz Biotechnology, Santa Cruz, CA, USA); anti‐FSHR (follicle‐stimulating hormone receptor; bs‐20658R‐AF488, Bioss, Beijing); anti‐actin (A5441; Sigma Chemical Co, St Louis, MI, USA); anti‐GAPDH (AP0066, Bioworld technology, co.Ltd); anti‐mouse and anti‐rabbit (PI‐2000 and PI‐1000; Vector Laboratories, CA, USA); Alexa Pluer™ 594 donkey anti‐rat lgG(H + L) (A‐21209; Thermo Fisher Scientific); CoraLite594‐conjugated Goat Anti‐Mouse IgG(H + L) (SA00013‐3, proteintech, USA); CoraLite594‐conjugated Goat Anti‐Rabbit IgG(H + L) (SA00013‐4, proteintech, USA); Goat anti‐Rabbit IgG (H + L) Cross‐Adsorbed Secondary Antibody, Alexa Fluor 647 (A‐21244, Thermo Fisher Scientific); Phosphate buffer solution (PBS; CC0002， Leagene Biotechnology, Beijing); pregnant mare serum gonadotropin (PMSG; Ningbo Second Hormone Factory, China); human chorionic gonadotropin (hCG; Ningbo Second Hormone Factory, China); and DAPI (Thermo Fisher Scientific). Both PMSG and hCG were dissolved in PBS and prepared to working concentration. Recombinant mouse KS protein was gifted by Prof. Guoquan Gao.^14^

### 2.2 Isolation of human primary granulosa cells

Human primary granulosa cells (PGCs) were isolated from the follicular fluids in 30 patients who received in vitro fertilization and embryo transfer (IVF‐ET) treatment in the Third Affiliated Hospital of Guangzhou Medical University as described previously.^15^, ^16^ First, the follicular fluids were collected and centrifuged at 526 *g* for 15 min (Sorvall ST40R; Thermo Fisher Scientific). The supernatant was discarded, and the pellet was added into the lymphocyte separation buffer (Biosharp Life Sciences, Hefei, Anhui, China) at a volume ratio of 1:2, followed by centrifugation at 234 *g* for 10 min. After that, the pellet was divided into four layers—follicle fluid layer, granulosa cell layer, lymphocyte separation medium layer and red blood cells layer. The granulosa cell layer was extracted and centrifuged at 234 *g* for 5 min with red blood cell lysis buffer (Biosharp Life Sciences) at a volume ratio of 1:3. The supernatant was discarded, and the pellet was washed thrice with PBS. The isolated granulosa cells were subjected to subsequent treatments. In all the experiments, only the first passage of PGCs was used.

All of the procedures in the present study were reviewed and supported by the Ethic Committee of Guangzhou Medical University. We received agreement, permission and signed consent from all patients included in the present study.

### 2.3 Determination of PGSs purity by flow cytometry

10 × 10^4^ PGCs were taken into a clean EP tube. Adding an appropriate amount of 4% paraformaldehyde to fix the cells and let them stand at room temperature for 15 min. Wash the cells, permeabilize the cell membrane with 0.3% Triton‐X 100, and stand at room temperature for 10 min. After washing and centrifugation, FSHR antibody was diluted with 5% BSA at the concentration of 1:100, and the cells were incubated in dark for 1 h. Flow cytometry was performed after washing. Cytoflex S.4 (Beckman, USA.) was used.

### 2.4 Cell culture

The KGN granulosa cell line was gifted by Prof. Jianqiao Liu.^17^ Human PGCs and KGN cells were cultured in DMEM/F12 or DMEM supplemented with 10% foetal bovine serum (FBS), penicillin (100 IU/ml) and streptomycin (100 μg/ml) at 37°C in a humidified atmosphere with 5% CO2.

### 2.5 Quantitative real‐time PCR (qPCR)

Total RNA in cells was extracted by total RNA column extraction kit (M050, New Cell&Molecular Biotech Co., Ltd). The concentration of extracted RNA was determined by nucleic acid quantitative instrument (ND2000, Thermo Scientific). The concentration and purity of RNA were measured with sterile water as the calibration. The purity was based on the value of OD260/OD280. Briefly, 500 ng total RNA was used for reverse transcription (RR036A, Takara Biomedical Technology Beijing Co., Ltd. Beijing) and qPCR (RR820A, Takara Biomedical Technology Beijing Co., Ltd. Beijing). Target mRNA was determined using the ABI Prism 7900 sequence PCR machine (Applied Biosystems, Foster City, CA, USA). β‐actin was used as the internal control. The primers' sequences were listed in Table 1.

**TABLE 1:** QPCR primers

Primer	Sequences
KS F	TCAAAGCCCTGTGGGAGAAACC
KS R	GGTTAGGGAGAATGAAAAACACGGT
VEGF F	GGCCTCCGAAACCATGAACT
VEGF R	CTGGGACCACTTGGCATGG
β‐actin F	GCACTCTTCCAGCCTTCCTT
β‐actin R	GTTGGCGTACAGGTCTTTGC

### 2.6 Western blotting analysis

Cells or tissues were lysed with 1× SDS lysis buffer (PS0015, LEAGENE, Beijing Leagene Biotechnology Co., Ltd) which is composed of Tris HCl, NaCl, SDS as well as a variety of protease inhibitors and so on. Total protein concentration was determined by using Bio‐Rad protein assay kit (Bio‐Rad, Hercules, CA, USA) according to the manufacturer's protocol. Equal amounts of cell extracts were subjected to electrophoresis on a 12% SDS‐PAGE (P0012A, Beyotime Biotechnology) and then transferred onto a polyvinylidene difluoride membrane (Immobilon‐PSQ Transfer Membrane, ISEQ00010 PORE SIZE:0.2 μm). After 3 h, the membrane was blocked using 7% non‐fat milk (1172, BioFroxx, Guangzhou saiguo biotech Co., Ltd) in Tris‐buffered saline (T8060, Solarbio, Beijing Solarbio Science&Technology Co., Ltd) with Tween‐20 (T8220, Solarbio, Beijing Solarbio Science&Technology Co., Ltd) for 1 h. The target blot was probed with antibodies against KS, VEGF and β‐actin. Bound antibodies were visualized using HRP‐conjugated secondary antibodies. The protein binding antibody was exposed under the action of luminescent solution (P0018S, Beyotime Biotechnology.), and ChemiDoc‐It Imaging System was used.

### 2.7 Enzyme‐linked immunosorbent assay (ELISA)

The levels of KS and VEGF in cell supernatant were measured with commercially available KS and VEGF ELISA kits (DY1669/DY293B‐05, R&D, Bio‐Techne China Co. Ltd). All procedures were performed according to the manufacturer's instructions.

### 2.8 Immunocytochemistry

Circular cell climbing sheets were placed in 24‐well plates (801,010, NEST, Jiangsu) in advance. Then, 8 × 10^3^ cells were inoculated into each well. Cells were washed after adhesion and fixed at room temperature with 4% paraformaldehyde (DF0135, Leagene Biotechnology, China) for 20 min. After washing 3 times for 5 min each time, cells were permeabilized with 0.1% Triton‐X 100 (abs9149, absin, Shanghai) and incubated at room temperature for 10 min. Cells were sealed with 2% BSA at room temperature for 1 h. After washing 3 times for 5 min each time, the antibody was diluted in 2% BSA at 1:100, added into the small hole and incubated overnight. The next day, the secondary antibody diluted at 1:200 was incubated in dark for 3 h at room temperature. After washing 3 times for 5 min each time under avoiding light, anti‐fluorescent quencher (P36965, ProLong™ Diamond, Invitrogen) which contains DAPI dye was added dropwise to the common glass slides. In addition, the circular cell climbing slides were buckled on them. The pictures were taken by confocal microscope (LSCM, Nikon, Japan). The fluorescence results of each channel were adjusted to an appropriate wavelength range to reduce the background. All fluorescence channels were finally superimposed, and then, TIFF format results were output in each channel. All results should be scaled to indicate the same field of view and focal length. All the images are stacked.

### 2.9 Animals

ICR mice were derived from Swiss mouse population and cultivated by the Institute of Cancer Research. Other countries introduced it and called ICR mouse. ICR mice have strong adaptability and fecundity, fast growth speed and good experimental repeatability. They are international closed colony mice and are widely used in the fields of life science, neuroscience, physiological psychology and pharmacology. So, we used ICR mice as experimental animals. Female ICR mice (Center for experimental animals in Guangdong Province, China) were housed in the air‐conditioned, light‐controlled, animal facility of the Guangzhou Medical University. Animal care was provided in accordance with the Institutional Animal Care and Use Committee guidelines.

### 2.10 OHSS mouse model

OHSS mouse model was established as described previously.^18^ PMSG (20 IU/d) was intraperitoneally injected into 5‐week‐old female ICR mice for three consecutive days to promote plenty of follicular development. Next, 7IU hCG was administered after the last injection of PMSG 24 h later to induce ovulation and ovarian hyperstimulation. The signs of enlargement of ovaries and increasing vascular permeability indicate the OHSS model is successfully constructed.

### 2.11 Evaluating the effect of KS on OHSS mice

Five‐week‐old female ICR mice were randomly divided into three groups. Each group had 8 mice. Group 1 (control) received a single dose intraperitoneally injection of 5 IU PMSG on day 2 and 7 IU hCG 48 h later. PBS was injected on Days 1 and 3. Group 2 (OHSS) was constructed as above described. Group 3 (OHSS + KS) was treated as the OHSS group, followed by intraperitoneally injected KS (2 mg/kg) instead of PBS. Body weight, ovarian weight and vascular permeability were assessed to determine the effect of KS.

### 2.12 Anti‐VEGF treatment

In order to study the role of VEGF in OHSS model, we designed VEGF neutralization experiment. The experiment procedure was the same as that of KS treatment. VEGF neutralizing antibody (AF‐493‐NA, R&D, Bio‐Techne China Co. Ltd) and corresponding negative control (AB‐108‐C, R&D, Bio‐Techne China Co. Ltd) were used in this experiment. The changes of body weight, ovarian weight and vascular permeability were also assessed.

### 2.13 Body and ovary weight measurement

We measured the weight of each mouse every day throughout the experiment. The increased weight was calculated between Day 1 and Day 6 and compared among groups. The mice were sacrificed by cervical dislocation after anaesthetised with 1.5% pentobarbital sodium, and the ovaries dissected for weighing and photographing (Canon, Japan). The weighing instrument we used was the electronic balance of METTLER TOLEDO instrument (Shanghai) Co., Ltd.

### 2.14 Permeability assay

Permeability was quantified by the modified Miles vascular permeability assay.^19^ Briefly, mice were anaesthetised with 1.5% pentobarbital sodium and injected with Evans blue dye (5 mM, 0.1 ml; Sigma Chemical Co) into caudal vein. After 30 min, we injected 2 ml sterile saline into the abdominal cavity of the mice and gently massaged the entire abdomen for 1 min. Then, we opened the abdominal cavity at the midline and extracted 1 ml fluid with a pipette. The fluid was centrifuged at 160 *g* for 5 min (TG16‐WS; xiangyi centrifuge instrument CO.,Ltd; China), and 200 μl supernatant was pipetted into 96‐well plates. The optical density (OD) values were measured at 405 nm (for calibration) and 620 nm, respectively.

### 2.15 Ovulation assay

Seven‐week‐old female ICR mice were randomly divided into two groups with 8 mice in each group. All mice were intraperitoneally injected with 5 IU PMSG for follicular development, and 7 IU hCG was administrated 48 h later to induce oocyte maturation and ovulation. Mice were intraperitoneally injected with KS (2 mg/kg) or PBS on the day of PMSG administration, respectively. Mice were sacrificed by cervical dislocation after anaesthetised with 1.5% pentobarbital sodium in 16–18 h after hCG administration. Ovulated, cumulus‐enclosed oocytes were isolated from the oviduct ampullae into M2 medium. Cumulus cells were removed by brief exposure to 400 IU/ml hyaluronidase (Sigma). The oocytes were observed under microscope (Nikon, Japan) and photographed. The number of oocytes and oocyte maturation rate (number of mature oocytes / total number of oocytes per mouse) was recorded.

### 2.16 Pregnancy and embryonic development assay

Seven‐week‐old mice were randomly divided into two groups with 6 mice in each group. All female ICR mice were intraperitoneally injected with 2 IU PMSG for inducing follicular development. In addition, 48 h later, 7 IU hCG was administrated. Then, the female ICR mice were allowed to mate overnight with males of proven fertility. Mating was verified by the presence of a vaginal plug. KS (2 mg/kg) or PBS was administered on the day of hCG administration and 3 days later. Mice were sacrificed by cervical dislocation after anaesthetised with 1.5% pentobarbital sodium on Day 16 after hCG administration. The abdominal cavity at the midline was opened, and foetal mice were removed from the uterus. Then, we recorded the number and weight of foetal mice. The representative foetal mice were photographed by camera (Canon, Japan).

### 2.17 Tissue immunofluorescence

Mice ovaries were fixed in 4% paraformaldehyde at room temperature and embedded with 4°C OCT (BL557A, biosharp, Hehui, Anhui, China) for producing frozen sections (5‐μm thick). Frozen sections were washed twice with PBS and fixed in 4°C acetone for 10 min. After that, the frozen sections were blocked with 2% bovine serum albumin (BSA), after which the sections were stained with anti‐VEGF antibody, secondary fluorescent antibody, and anti‐fluorescent quencher containing DAPI dye and photographed under a laser scanning confocal microscope (LSCM, Nikon, Japan).

### 2.18 Statistical analysis

All data were repeated 3 times in the same continuous time. Data are represented as mean ± SEM. All statistical analyses were performed using GraphPad Prism version 5. We used Image J to semi‐quantitatively analyse the results of Western blotting and cellular immunofluorescence. The protein bands in the Western blotting results were transformed into grey absorption peaks in Image J. We circled all the peaks and obtained the total area of the absorption peaks. Then, we compared the target genes with the internal reference to obtain the grey analysis results. Immunofluorescence images were processed in Image J and transformed into the proportion of fluorescence region and fluorescence intensity per unit area. The two groups were compared using Student's t‐test. *p* < 0.05 was considered statistically significant.

## 3 RESULTS

### 3.1 HCG can down‐regulate KS expression and up‐regulate VEGF expression

**FIGURE 1:**
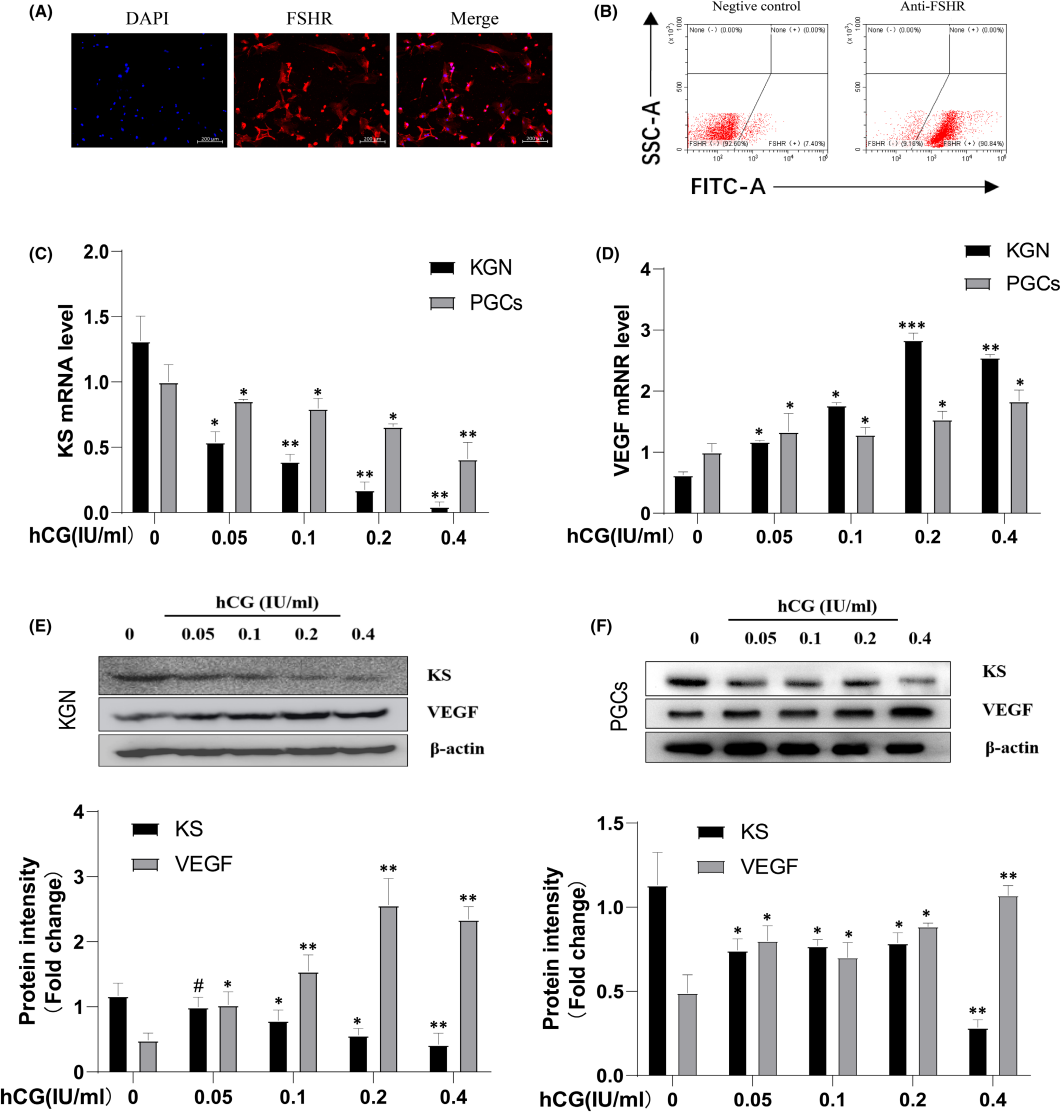
Inverse regulation of hCG on KS and VEGF expression in KGN cells and human PGCs. (A and B) Immunofluorescence and flow cytometry were used to evaluate the purity of PGCs. KGN cells and PGCs were serum‐starved for 16 h and then stimulated with gradually increasing concentrations of hCG from 0.05 to 0.4 IU/ml for 8 h. QPCR and Western blotting were employed to measure the mRNA (C and D) and protein (E and F) levels of KS and VEGF, respectively. The KS and VEGF protein level were semi‐quantified by densitometry and normalized relative to the β‐actin levels by Image J software. Quantitative data are presented as mean ± SEM. *p < 0.05; **p < 0.01.

FSHR is a specific marker of granulosa cells, which was commonly used to determine the purity of granulosa cells.^20^, ^21^ Immunofluorescence microscopy showed that almost all of isolated cells expressed FSHR (Figure 1A). Moreover, flow cytometry assay showed that the purity of PGCs was more than 83% compared with the negative control (Figure 1B). These results suggested that our purification method obtained a high percentage of human PGCs that can be used for the subsequent experiments.

As hCG is the pivotal trigger of OHSS,^4^ we evaluated its effect on KS and VEGF expression at different time points. In KGN cells, the secretion, mRNA and protein levels of KS and VEGF can be regulated after hCG treating for 8 h or 12 h, but not 4 h (Figure S1A,B,E,F,I,J). In PGCs, hCG can regulate KS and VEGF at all time points (Figure S1C,D,G,H,K,L). Based on these results, we chose the time point of 8 h. At this time point, hCG decreased the expression of KS mRNA in KGN cells and PGCs in a dose‐dependent manner (Figure 1C). Inversely, VEGF, which was supposed to be a key inducer of OHSS development,^5^ was found to be up‐regulated at the mRNA level by increasing hCG concentration (Figure 1D). Furthermore, the changed trend of KS and VEGF at the protein level was similar to that of mRNAs (Figure 1E,F). These results indicate that hCG may be the main cause of imbalance of KS and VEGF expression in OHSS.

### 3.2 KS decreases VEGF expression

**FIGURE 2:**
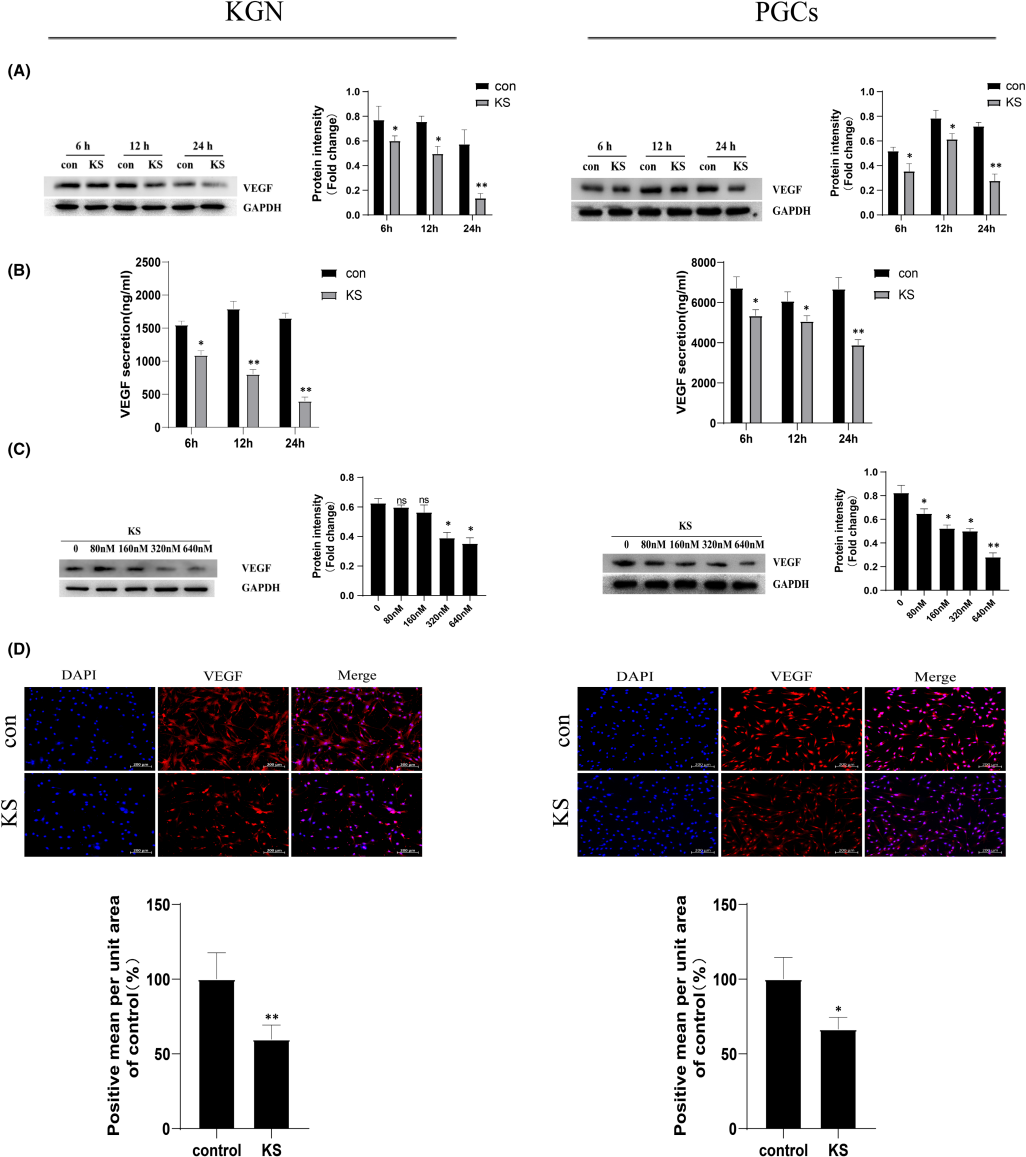
KS down‐regulates VEGF expression in KGN cells and human PGCs. KGN cells and PGCs were stimulated with recombinant KS protein in 640 nM for 6, 12 and 24 h. The cells and cell‐free supernatants were subjected to Western blotting (A) and ELISA (B), respectively. (C) KGN cells and PGCs were stimulated with gradually increasing concentrations of KS from 80 to 640 nM for 24 h. Western blotting assay was performed. (D) KGN cells and PGCs were exposed to 640 nM KS for 24 h. Cells were stained with VEGF fluorescent antibody (red) and DAPI (blue). Shown are representative pictures from three independent experiments. Scale bars: 200 μm. The protein level and fluorescence were semi‐quantified by Image J software. Quantitative data are presented as mean ± SEM. *p < 0.05; **p < 0.01. con: control. ns: not significant.

To investigate the effect of KS on VEGF expression in vitro, KGN cells and human primary granulosa cells were used. Our findings showed that 24‐h treatment with KS resulted in most significant down‐regulation of VEGF expression and secretion in both KGN cells and PGCs (Figure 2A,B). Therefore, we evaluated the dose‐dependent effect of KS at the time point of 24 h. With the dose increasing, the secretion and protein level of VEGF showed a decrease trend. The inhibitory effect of KS on VEGF expression was most significant at the concentration of 640 nM (Figure 2C). By treatment with this concentration, we also observed an obvious attenuation of red fluorescence both in KGN cells and in PGCs (Figure 2D). These data suggest that KS can inhibit VEGF expression in the granulosa cell model.

### 3.3 KS prevents OHSS by reducing vascular leakage

**FIGURE 3:**
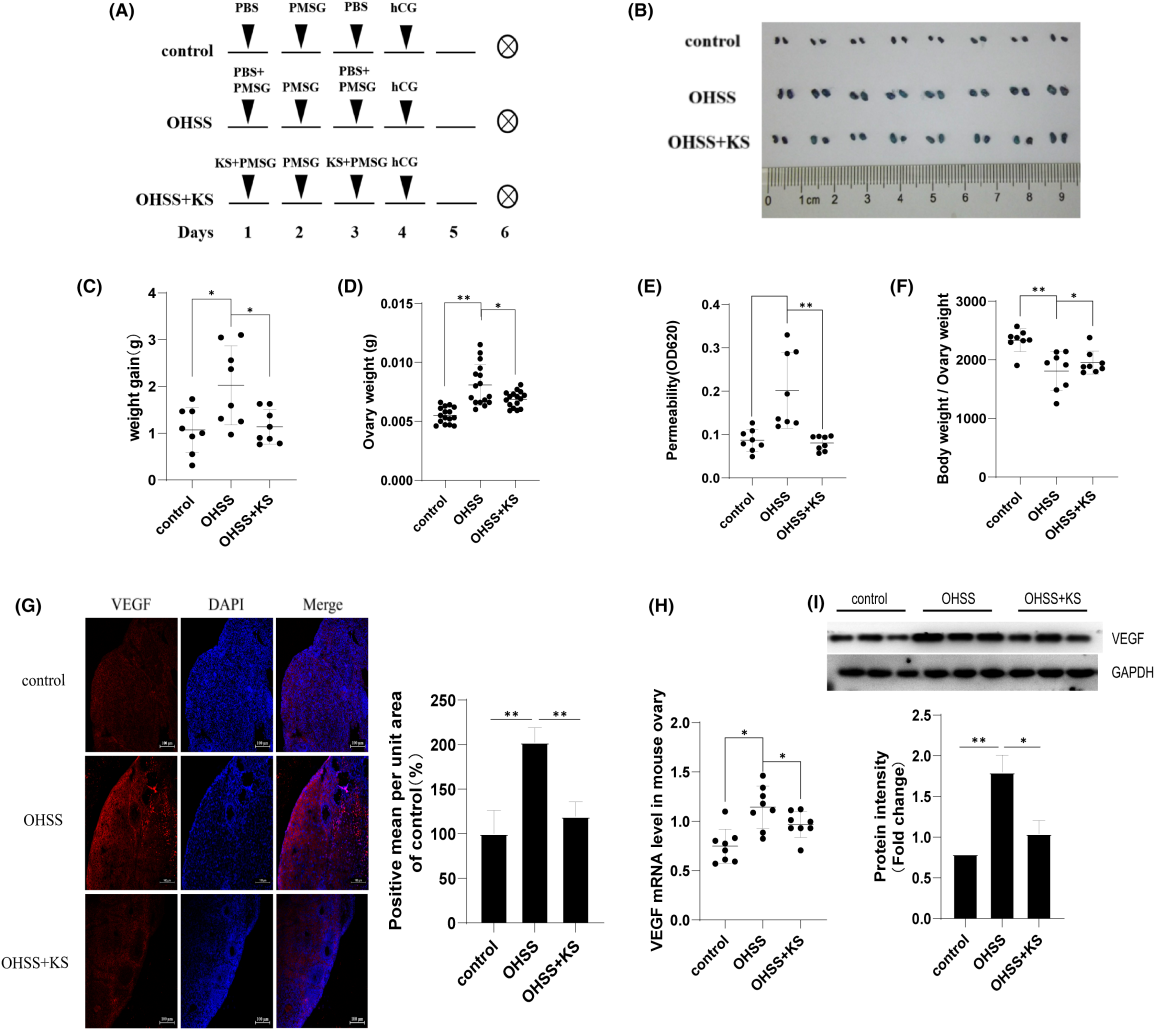
Subcutaneous administration of KS protein alleviated OHSS signs by regulating vascular leakage. (A) Schematic representation of the experimental setting. (B) Typical photographs of ovaries. Weight gain (C), ovarian weight (D) and vascular permeability (E) were assessed. (F) Comparison of the expression of VEGF in ovaries by immunofluorescence among the control group, OHSS group and OHSS+KS group. The VEGF protein is shown in red and the nucleus is blue. Shown are representative pictures from three independent experiments. Scale bar: 100 μm. (G) The levels of VEGF mRNA in ovarian tissues were evaluated using qPCR. (H) The levels of VEGF protein in ovarian tissues were evaluated using Western blotting. (I) The protein level and fluorescence were semi‐quantified by Image J software. *N* = 8 mice per group. Data are shown as mean ± SEM. *p < 0.05; **p < 0.01.

In order to confirm the key role of VEGF in the pathogenesis of OHSS in vivo, we carried out VEGF antibody neutralization experiment (Figure S2A). Compared with the control group, OHSS mice had significantly increased body weight, ovarian weight and increased vascular permeability, while neutralizing VEGF alleviated these signs (Figure S2B‐F). Based on the above findings, an exogenous supply of KS protein may have an inhibitory effect on OHSS. To confirm this hypothesis, we designed the intervention experiment with KS (Figure 3A). As expected, OHSS mice gained significant body and ovary weight accompanied by increased vascular permeability, as compared with control mice; administration of KS alleviated these signs (Figure 3B‐E). Further, the ovaries were subjected to immunofluorescence and Western blotting. Compared with the control group, the OHSS group showed a marked increase in fluorescence intensity and protein level of VEGF, but these were reduced in the OHSS+KS group (Figure 3F,G).

### 3.4 KS has no effect on ovulation, pregnancy and embryo development

**FIGURE 4:**
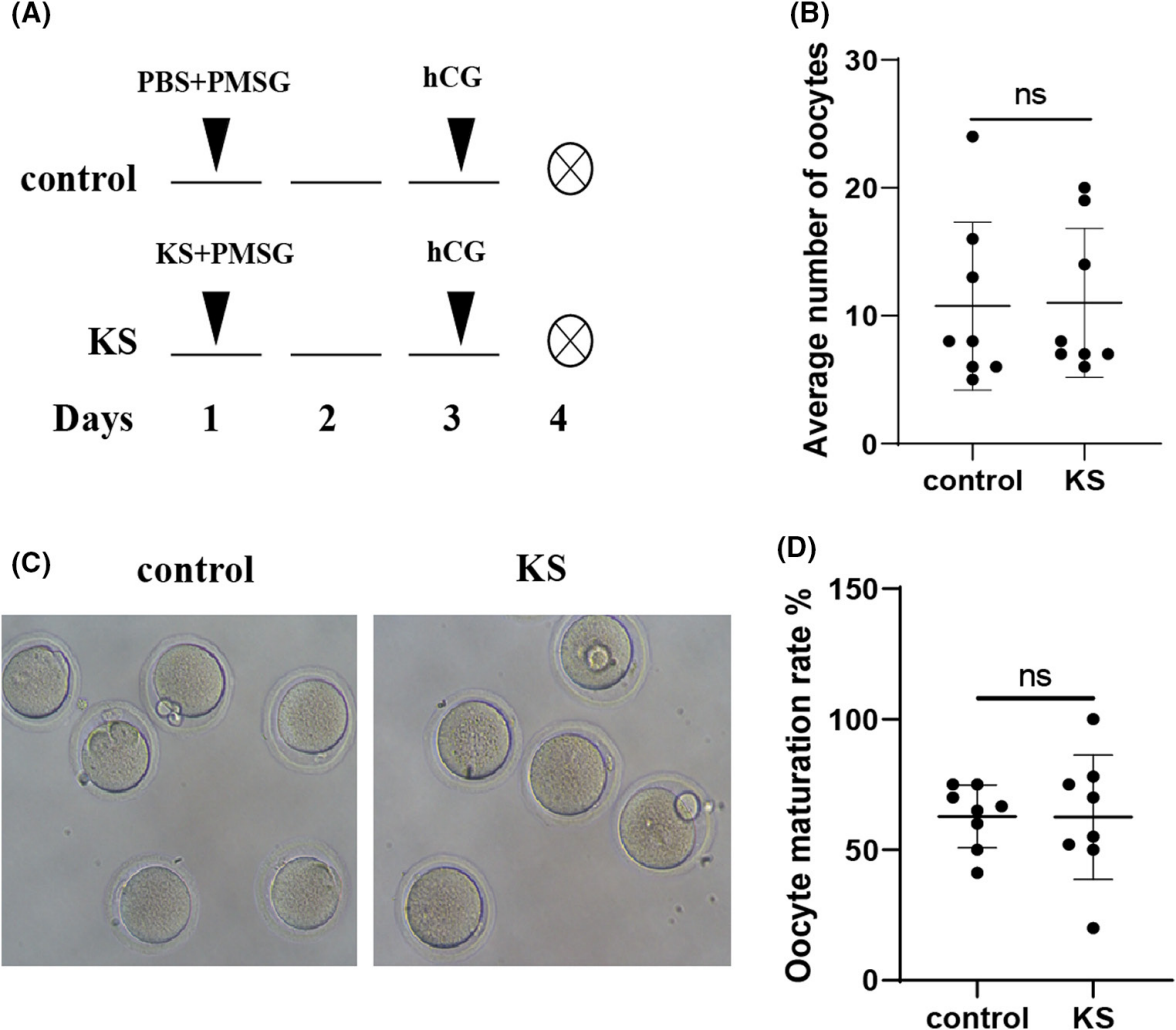
Effect of KS protein on ovulation. (A) Schematic representation of the experimental setting. The ovulated oocytes number (B), oocytes morphology (magnification: ×200) (C), and (D) oocyte maturation rate (the number of MII oocytes/the total number of oocytes per mouse) were compared between the control group and the KS group. *N* = 8 mice per group. Data are shown as mean±SEM. *p < 0.05; **p < 0.01. ns, not significant.

**FIGURE 5:**
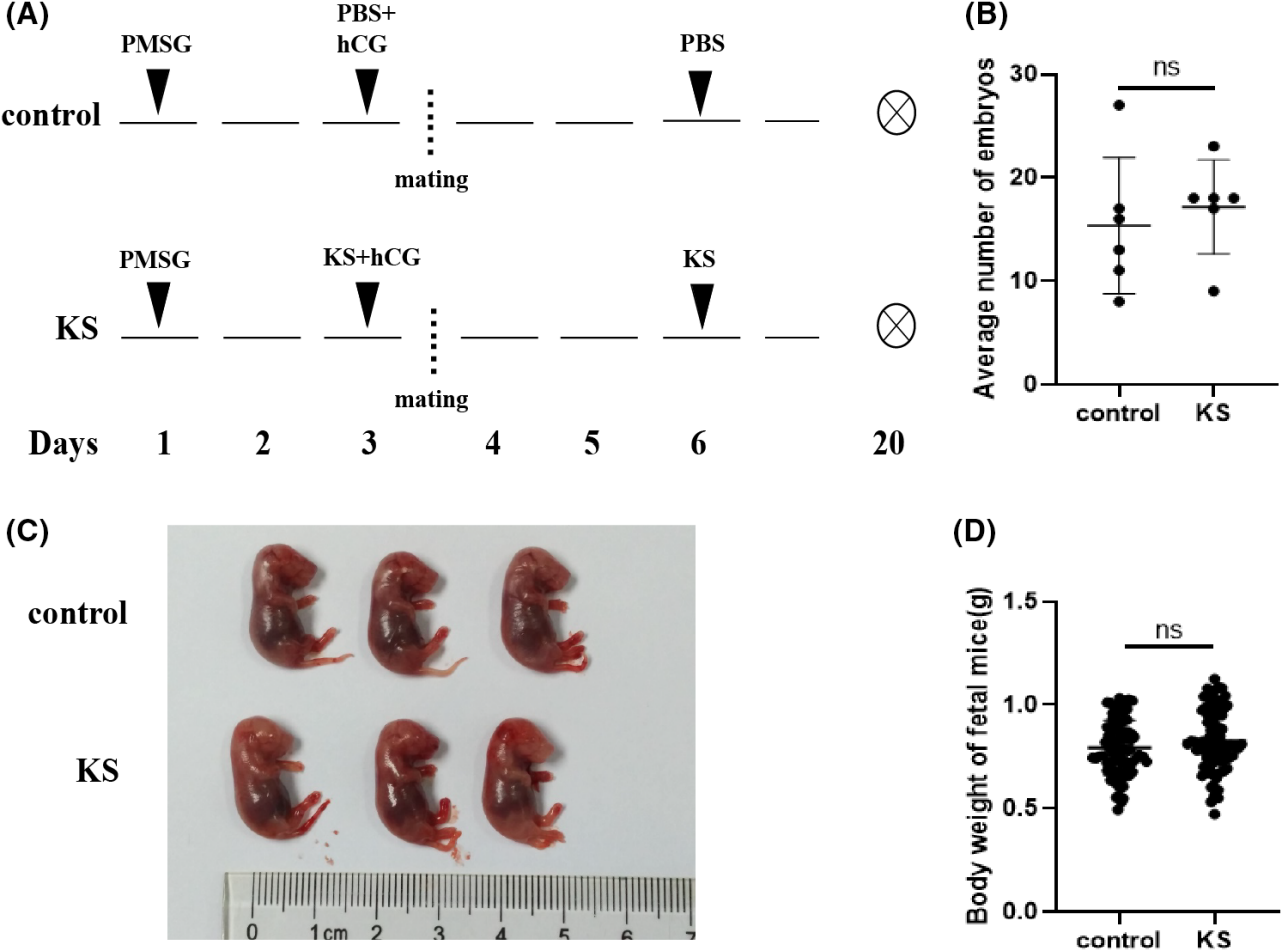
Effect of KS protein on pregnancy and fetus development. (A) Schematic representation of the experimental design. The number (B) and weight of mice fetuses (D) were assessed on Day 16 after hCG administration. (C) Typical photographs of the mice fetuses. *N* = 6 mice per group. Data are shown as mean ± SEM. ns, not significant.

We evaluated whether KS treatment had any side effects on reproduction, ovulation, pregnancy and embryonic development. The experiment procedure was shown as Figure 4A. There was no significant difference in the number of retrieved oocytes, oocyte maturation rate and oocyte morphology between the control and KS groups (Figure 4B‐D). Furthermore, we constructed a pregnant mice model using 2 IU PMSG and 7 IU hCG. PBS or KS was administrated on the day of hCG injection and 3 days later (Figure 5A). No significant inter‐group difference was found in the number of embryos or foetal development on Day 16 after hCG administration (Figure 5B‐D). Taken together, these results indicate that KS has no impact on reproduction.

## 4 DISCUSSION

Many studies have identified VEGF as a key inducing factor for the increase of vascular permeability, which leads to the occurrence and development of OHSS.^5^ In this study, we clarified that the level of VEGF was up‐regulated in mice with OHSS. The increase of VEGF was verified to be due to hCG treatment. Moreover, anti‐VEGF treatment attenuated OHSS signs in a mouse model.

Besides VEGF, the decrease of anti‐angiogenic factors was reported to be one of the mechanisms leading to the occurrence of OHSS.^18^ Therefore, we investigated whether the anti‐angiogenic factor KS was regulated by hCG. In our study, the expression of KS was found to be significantly down‐regulated in KGN cells and human PGCs in a dose‐dependent manner, which was the opposite in VEGF regulation by hCG. As mentioned above, VEGF expression could be inhibited by KS in various cell models.^9^, ^10^, ^11^, ^13^ We also obtained the same results in granular cells. These results implied that the reduced KS may fail to counteract the effect of high levels of VEGF and result in OHSS.

Several pathways and factors have been identified to mediate the modulation of VEGF by hCG, such as EGFR/HER2 signalling pathway and C/EBPβ.^22^, ^23^ To our best knowledge, as this appears to be first report of hCG regulation of KS, the molecular mechanism of this regulation is still unclear. It is reported that the orphan nuclear receptor, COUP‐TF1, can reverse the effect of triiodothyronine on the down‐regulation of KS expression by binding to a negative thyroid hormone‐binding element.^24^ Another study demonstrated that the transcription factor FOXO1 mediated the down‐regulation of KS by hydrogen peroxide in endothelial cells.^25^ Moreover, hCG promoted the expression of FOXO1.^26^ Hence, we speculate that COUP‐TF1 and FOXO1 are likely to mediate the inhibitory effect of hCG on KS, which needs to be further identified.

Given that VEGF is the main protein participating in the pathophysiology of OHSS,^27^ and the level of VEGF could be down‐regulated by KS both in vitro and in animal models in our study, we assumed that KS prevents OHSS, at least in part, by inhibiting VEGF. Previous studies have shown that KS decreased VEGF expression via the Wnt signalling pathway.^13^, ^28^ Besides this, Notch signalling pathway was supposed to be involved in VEGF regulation.^29^, ^30^, ^31^ These studies indicated that KS may also function through the Notch signalling pathway. The exact mechanisms by which KS regulates VEGF expression need to be further investigated.

Since anti‐VEGF therapy was shown its efficacy in other diseases,^32^, ^33^, ^34^, ^35^, ^36^, ^37^, ^38^several compounds based the same mechanism have been suggested as potential therapeutic agents for OHSS. SU5416, a synthetic compound targeting VEGFR2, could restore the increased vascular permeability in hyperstimulated rats.^39^ Dopamine agonists, also targeting VEGFR2, were shown to reduce the incidence of OHSS in IVF cycles.^40^ Metformin was reported to be beneficial in reducing OHSS incidence among patients with polycystic ovarian syndrome.^41^ However, SU5416 displayed adverse effects on follicular development and pregnancy.^42^, ^43^ Dopamine agonists only worked in moderate OHSS, but not severe OHSS.^40^ Metformin had a limited application scope, being only effective in those with polycystic ovarian syndrome patients with high‐risk OHSS.^41^ In the present study, KS was proven to be effective in preventing OHSS and safe for ovulation, pregnancy and foetus development in mice.

## 5 CONCLUSION

In conclusion, the present study reveals that KS may be a novel potential drug candidate for preventing OHSS in clinical practice in which KS can suppress VEGF expression to decrease vascular permeability, and thus alleviated OHSS development without side effects on reproductive function of mice. However, there are several limitations in this study, such as lack of detailed mechanisms by which KS regulating VEGF and more comprehensive safety assessment of KS on reproductive function, which require further in‐depth research in the future.

## AUTHOR CONTRIBUTIONS

**JianFang Huang:** Data curation (equal); investigation (lead); methodology (lead); software (lead); writing – original draft (equal). **Yuling Mao:** Investigation (equal); methodology (equal). **Quanxin Li:** Investigation (equal); methodology (equal). **Honghai Hong:** Investigation (equal); methodology (equal). **Ni Tang:** Data curation (supporting); formal analysis (supporting). **Xiangjin Kang:** Conceptualization (supporting); methodology (supporting). **Yuling Huang:** Resources (supporting); software (equal). **Jianqiao Liu:** Conceptualization (supporting); funding acquisition (supporting); resources (equal). **Qin Gong:** Conceptualization (equal); resources (equal). **Yachao Yao:** Conceptualization (equal); writing – original draft (equal). **Lei Li:** Data curation (equal); funding acquisition (lead); project administration (lead); supervision (lead); writing – review and editing (lead).

## CONFLICT OF INTEREST

The authors declare that they have no conflict of interest.

## Supporting information


FigureS1

FigureS2

## Data Availability

The data that support the finding of this study are available from the corresponding author upon reasonable request.
