# Supplementary figures and images for: Kallistatin prevents ovarian hyperstimulation syndrome by regulating vascular leakage
Source: J Cell Mol Med. 2022 Jul 21;26(16):4613–23. doi: 10.1111/jcmm.17491 (PMC9357611; doi:10.1111/jcmm.17491)

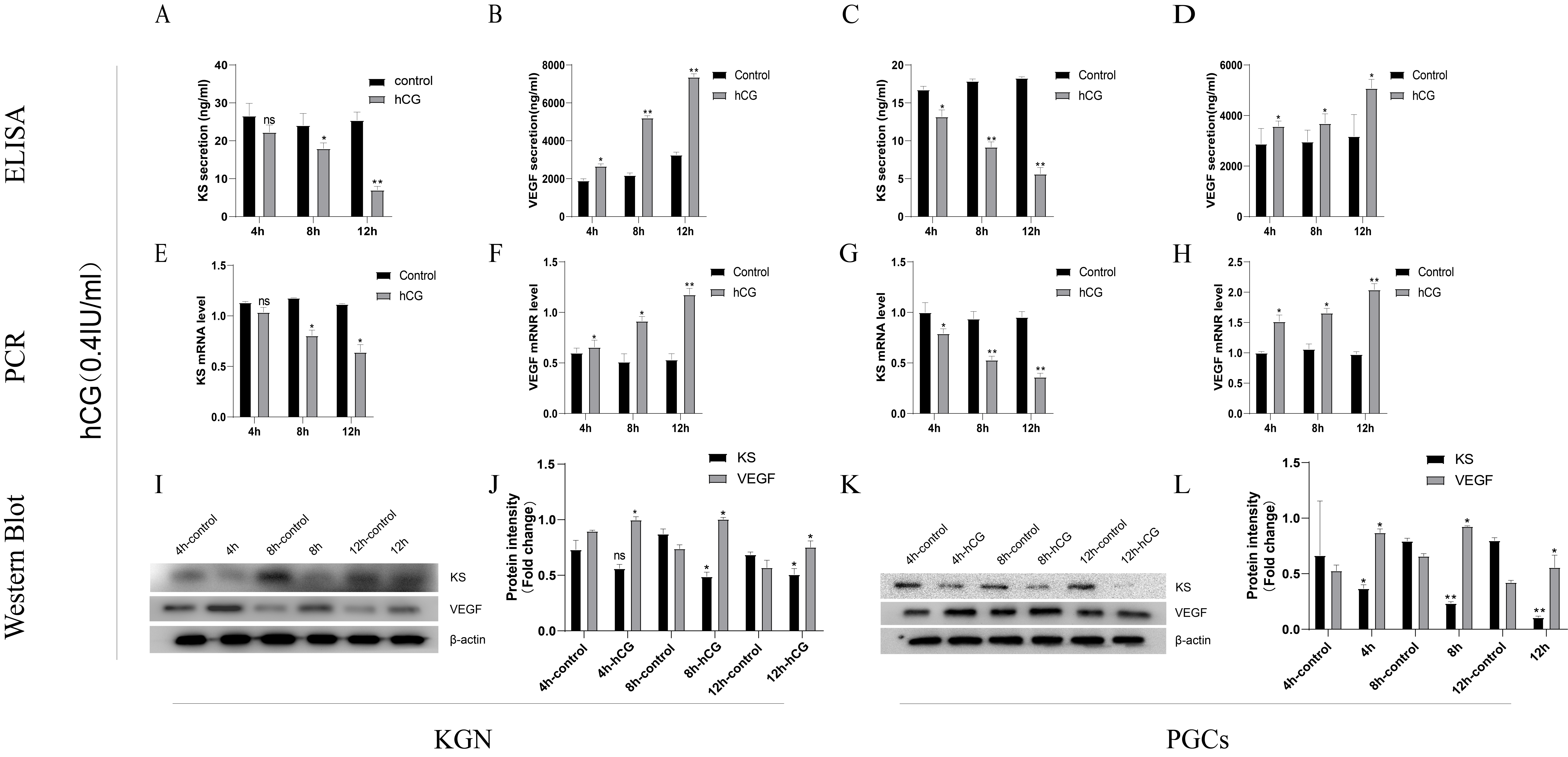

Supplement: Supplementary file 1 — FigureS1 [file JCMM-26-4613-s002.png]

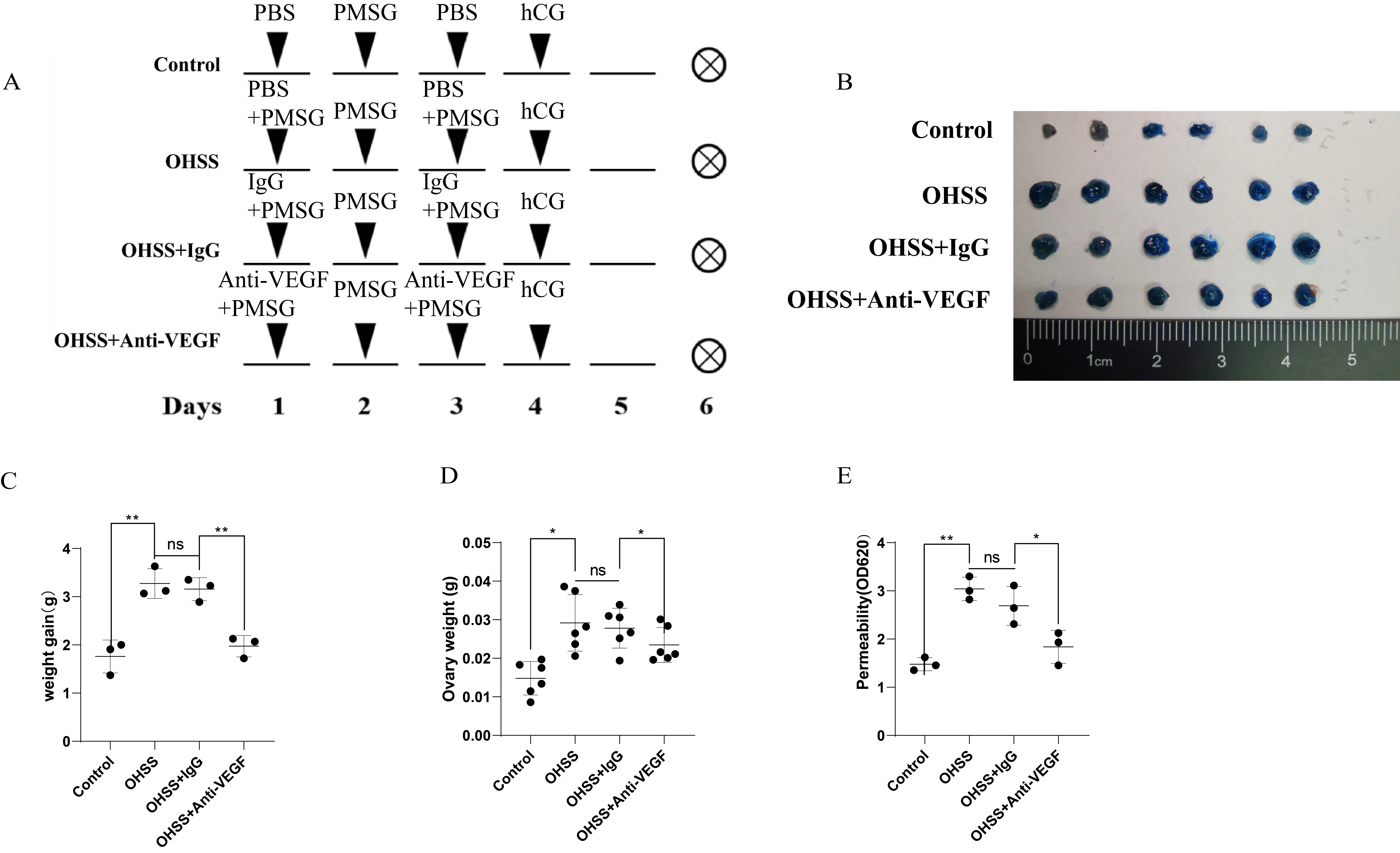

Supplement: Supplementary file 2 — FigureS2 [file JCMM-26-4613-s001.png]
